# Spinal cord regeneration in *Xenopus* tadpoles proceeds through activation of Sox2-positive cells

**DOI:** 10.1186/1749-8104-7-13

**Published:** 2012-04-26

**Authors:** Marcia Gaete, Rosana Muñoz, Natalia Sánchez, Ricardo Tampe, Mauricio Moreno, Esteban G Contreras, Dasfne Lee-Liu, Juan Larraín

**Affiliations:** 1 Center for Aging and Regeneration, Millennium Nucleus in Regenerative Biology, Faculty of Biological Sciences, Pontificia Universidad Católica de Chile, Alameda, 340, Santiago, Chile; 2 Faculty of Medicine, Pontificia Universidad Católica de Chile, Alameda, 340, Santiago, Chile

**Keywords:** Spinal cord regeneration, Sox2, *Xenopus*

## Abstract

**Background:**

In contrast to mammals, amphibians, such as adult urodeles (for example, newts) and anuran larvae (for example, *Xenopus*) can regenerate their spinal cord after injury. However, the cellular and molecular mechanisms involved in this process are still poorly understood.

**Results:**

Here, we report that tail amputation results in a global increase of Sox2 levels and proliferation of Sox2^+^ cells. Overexpression of a dominant negative form of Sox2 diminished proliferation of spinal cord resident cells affecting tail regeneration after amputation, suggesting that spinal cord regeneration is crucial for the whole process. After spinal cord transection, Sox2^+^ cells are found in the ablation gap forming aggregates. Furthermore, Sox2 levels correlated with regenerative capabilities during metamorphosis, observing a decrease in Sox2 levels at non-regenerative stages.

**Conclusions:**

Sox2^+^ cells contribute to the regeneration of spinal cord after tail amputation and transection. Sox2 levels decreases during metamorphosis concomitantly with the lost of regenerative capabilities. Our results lead to a working hypothesis in which spinal cord damage activates proliferation and/or migration of Sox2^+^ cells, thus allowing regeneration of the spinal cord after tail amputation or reconstitution of the ependymal epithelium after spinal cord transection.

## Background

In mammals, including humans, spinal cord injury (SCI) results in loss of motor and/or sensory function below the level of the injury, leading to paraplegia and quadriplegia, both highly prevalent conditions with significant impact on life quality. SCI results in a massive loss of local neurons at the site of the lesion without neural regeneration [1]. Unlike mammals, amphibians such as adult urodeles (for example, newts), anuran larvae (for example, *Xenopus*) and fish can regenerate their spinal cord after injury [2-4]. Therefore amphibians are an attractive model to understand the cellular and molecular mechanisms involved in spinal cord regeneration, and might provide valuable knowledge and novel insights into understanding why spinal cord regeneration is absent in mammals.

Amphibian regeneration has been studied using two experimental approaches: tail amputation and spinal cord transection. Tail amputation induces the regeneration of a new posterior half of the tail containing spinal cord, skeletal muscle, notochord, fins, vasculature and skin [3,5,6]. After spinal cord transection both *Xenopus laevis* and salamanders can re-establish nerve tracts and achieve functional recovery from paraplegia [6-8]. Interestingly, in *Xenopus* this ability is restricted to the larval stages and is lost at the end of metamorphosis [9,10].

After tail amputation ependymal cells migrate to the wound area sealing the damaged spinal cord canal tip, forming a terminal dilatation lined by ependymal epithelium. This structure is referred to as neural ampulla or terminal vesicle [3]. Subsequently, the neural ampulla is alongside a bullet-like structure corresponding to the proliferative cells from the notochord and undifferentiated mesenchymal cells which give rise to the regenerative bud. Lineage tracing analyses have demonstrated that skeletal muscle, spinal cord and notochord regenerate from their own original tissues on the regenerative bud [11], suggesting that tail regeneration occurs through the activation of tissue-specific stem and/or progenitor cells. Accordingly, it has been demonstrated that activation of muscle progenitors, Pax7^+^ satellite cells, are required for skeletal muscle regeneration, resembling cellular mechanisms involved in homeostatic and reparative regeneration in mammals [12].

Regarding spinal cord regeneration, lineage tracing experiments have shown that cells contained within 500 μm of the spinal cord located rostral to the amputation plane are sufficient to populate the entire regenerated spinal cord [13,14]. Ependymal cells lining the central canal rearrange after transection and seal off the rostral and caudal stumps of the transected spinal cord, and subsequently start to proliferate and migrate to fill in the gap between both stumps [6-8,15]. This bridge of ependymal cells provides the substrate for nerve fibers to grow and regenerate a functional spinal cord [7,15]. In addition, 5-bromo-2'-deoxyuridine (BrdU) labeling experiments have demonstrated that in urodeles, ependymal cells are able to originate new neurons [6,8,16].

The *SoxB1* family (Sox1, Sox2 and Sox3) of sex determining region Y (SRY)-related transcription factors is expressed in the epiblast and neuroectoderm during development, as well as in neural stem cells and neural progenitor cells (NSCs and NPCs, respectively), and ependymal cells in the neurogenic regions of the adult brain [17-19]. *SoxB1* members block cell cycle exit allowing maintenance of NSC/NPC identity and self-proliferation during development and adult neurogenic domains [18,20-24]. In addition, Sox1 and Nestin are upregulated in response to damage in urodeles [25,26].

In the present work, we studied the expression of Sox2 and the effect of the overexpression of a dominant negative form in tail and spinal cord regeneration in *Xenopus*. We found that Sox2^+^ cells are present in the ependymal zone of the spinal cord of *Xenopus* larvae. Tail amputation resulted in a global increase of Sox2 levels in the spinal cord, as well as proliferative activation of Sox2^+^ cells. Transgenesis using a predicted Sox2 dominant negative construct decreased proliferation of spinal cord resident cells and disrupted spinal cord and tail regeneration. These results suggested that members of the SoxB1 family (Sox1-3) may be necessary for proper spinal cord and tail regeneration. Furthermore, Sox2 levels correlated with regenerative capabilities throughout metamorphosis. After transection, Sox2^+^ cells lining the ependymal canal might migrate to the ablation gap forming aggregates in order to restore the continuity of the spinal cord. These data support a model in which spinal cord injury activates proliferation of Sox2^+^ cells to allow the growth of a new spinal cord after tail amputation and the reestablishment of the spinal cord continuity in the ablation gap after transection.

## Results

### Sox2 expression levels increase in response to tail amputation in *Xenopus* tadpoles

**Figure 1:**
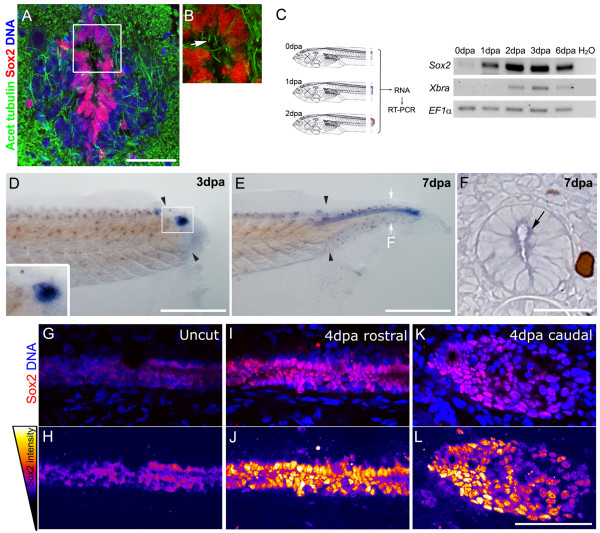
**Sox2 is upregulated after tail amputation. (A,B)** Immunofluorescence of transverse sections of stage 49 tadpoles with anti-Sox2 (red) and anti-acetylated α-tubulin (green) antibodies; a 20 μm Z-projection was made to detect cilia (arrow). DNA was counterstained with TOTO3 (blue). (B) Magnification of the inset indicated in (A). **(C)** Reverse transcription polymerase chain reaction (RT-PCR) analysis of *sox2* and *Xenopus brachyury* (*Xbra*) mRNA levels at the tip of the regenerating tail indicating that *sox2* is upregulated after tail amputation. *EF1α* was used as loading control. **(D-F)***In situ* hybridization using an antisense probe against *sox2* during tail regeneration of stage 49 tadpoles at (D) 3, and (E,F) 7 days post amputation (dpa), *sox2* was detected on the spinal cord and (D, inset) neural ampulla. (F) Transversal section of the regenerating tail at 7 dpa at the level indicated in (E), arrow indicates *sox2* staining in the ependymal epithelium. **(G-L)** Sagittal optical section of whole-mount immunofluorescence with anti-Sox2 antibodies (red) before amputation (G,H) and at 4 dpa (I,J) rostral and (K,L) caudal regions. DNA was stained in blue. (H,J,L) Pseudocolored intensities of Sox2 channel from (G), (I) and (K), respectively as is described in the left scheme. Arrowheads: amputation plane. Scale bars: (A) 25 μm, (D,E) 400 μm, (F-L) 50 μm.

Ultrastructural studies have demonstrated the presence of ciliated ependymal cells within a pseudostratified ventricular layer in *Xenopus* larvae spinal cord [15]. To determine if these cells correspond to neural progenitors, we evaluated Sox2 and acetylated α-tubulin expression using immunofluorescence on spinal cord cryosections from stage 49 tadpoles. We found that cells in the ependymal tube showed nuclear localization of Sox2, have elongated nuclei and are ciliated, all hallmarks for neural progenitors (Figure 1A,B; arrow). It is worth noting that these results suggest a dorsal-to-ventral gradient with higher levels of Sox2 protein in the ventral part of the ependymal tube.

To study the expression of Sox2 during tail regeneration, we evaluated the effect of tail amputation on *sox2* mRNA and protein levels. To accomplish this, we amputated stage 49 tadpole tails, isolated RNA from the tip of the regenerating tail at different days post amputation (dpa), and performed reverse transcription polymerase chain reaction (RT-PCR) analysis. Basal levels of *sox2* were detected immediately after amputation (0 dpa; Figure 1C). These levels were upregulated over time after amputation (Figure 1C). We observed a similar upregulation for *Xenopus Brachyury* (*Xbra*) mRNA, although delayed when compared to *sox2* (Figure 1C).

To determine the specific localization of *sox2* mRNA we performed whole-mount *in situ* hybridization in regenerating tails. At 3 dpa *sox2* was expressed in the neural ampulla at the distal tip of the regenerating tail (Figure 1D). At 7 dpa, *sox2* mRNA localizes in a cord-like structure along the regenerated tail (Figure 1E). Sections at 7 dpa showed that *sox2* mRNA is located in the spinal cord tissue (Figure 1F arrow and Additional file 1A).

In agreement with *sox2* mRNA expression, whole-mount immunofluorescence detected low levels of Sox2 protein in uncut tails (Figure 1G,H). A strong increase in Sox2 protein levels and Sox2^+^ cells was detected at 4 dpa in the spinal cord rostral to the amputation site, and in the neural ampulla at the tip of the regenerating tail (Figure 1I-L and Additional file 1B,C). Cells in the neural ampulla were ciliated giving further support to their neural progenitor characteristics ( Additional file 1D). After amputation, the rostral spinal cord contained multiple layers of Sox2^+^ cells (Figure 1I,J) and some peripherical nucleus display delamination-like morphology (see arrows in Additional file 1E). In summary, tail amputation increased Sox2 protein and mRNA levels as well as the number of Sox2^+^ cells in the spinal cord.

### Systemic upregulation of Sox2 levels after tail amputation

**Figure 2:**
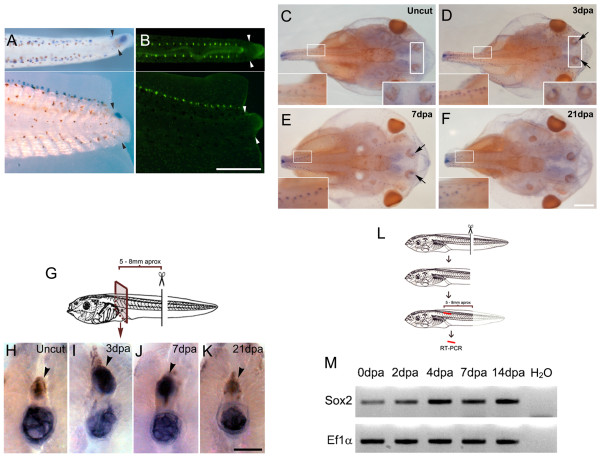
**Systemic upregulation of Sox2 after tail amputation. (A,B)** Tails from stage 49 tadpoles at 3 days post amputation (dpa). Dorsal (top) and lateral (bottom) view. (A) *sox2 in situ* hybridization and (B) 2-(4-(dimethylamino)styryl)-*N*-ethylpyridinium iodide (DASPEI) vital dye staining to identify the lateral line. Arrowheads indicate the amputation plane. **(C-F)** Dorsal view of *sox2* whole mount *in situ* hybridization of the cephalic region of (C) non-amputated tadpoles, and tadpoles at (D) 3, (E) 7 and (F) 21 dpa. Insets show higher magnification of the lateral line and olfactory epithelial region that increase *sox2* expression at 3 and 7 dpa. Notice that after fixation, tails were removed from their base and the observed stump ends do not correspond to the original amputation. **(G)** Diagram depicting the experimental set up for (H-K). **(H-K)***sox2 in situ* hybridization in areas 5 to 8 mm rostral to the amputation plane from tadpoles at different dpa. *sox2* is present in the spinal cord at 3 and 7 dpa (indicated by arrowheads). Endogenous expression of alkaline phosphatase in the notochord has been described previously [27] suggesting that expression on this tissue is non-specific. **(L)** Diagram depicting the experimental set up for (M). **(M)** Reverse transcription polymerase chain reaction (RT-PCR) analysis of *sox2* mRNA levels in isolated spinal cord indicates that *sox2* is upregulated in a rostral region during tail regeneration. *EF1α* was used as loading control. Scale bars: (A,B) 400 μm, (C-F) 400 μm, (H-K) 100 μm.

During *sox2* expression analysis we observed that tail amputation also upregulated *sox2* mRNA levels in specific cellular structures located dorsal and bilateral to the spinal cord (Figure 2A). These structures correspond to neuromasts of the lateral line as demonstrated by the similar staining pattern obtained with the vital dye 2-(4-(dimethylamino)styryl)-*N*-ethylpyridinium iodide (DASPEI) (Figure 2B). A transient increase of *sox2* expression was also detected in the anterior lateral line and the olfactory epithelial region at 3 and 7 dpa (see insets in Figure 2C-E). At 21 dpa, *sox2* mRNA returned to basal levels (Figure 2F).

This prompted us to investigate whether *sox2* levels in the spinal cord were also affected distantly to the amputation site. Non-amputated larvae and larvae at different dpa were fixed, cut 5 to 8 mm rostral to the amputation site, and *sox2* whole-mount *in situ* hybridization was performed (Figure 2G). At 3 and 7 dpa a transient upregulation of *sox2* mRNA levels was found in the spinal cord rostral to the amputation site (Figure 2H-K, compare staining levels in the spinal cord indicated by an arrowhead). Similar results were also obtained when the levels of *sox2* mRNA were evaluated by RT-PCR on isolated spinal cord rostral to the amputation plane (Figure 2L,M). Similar *sox2* mRNA levels were observed on isolated spinal cord during normal development at stages 50 and 56 (see Additional file 1F). Therefore, tail amputation resulted in a systemic upregulation of Sox2 in the spinal cord, the lateral line and the olfactory epithelium region, as part of a general response of the organism to tissue injury.

### Tail amputation activates proliferation of Sox2^+^cells

**Figure 3:**
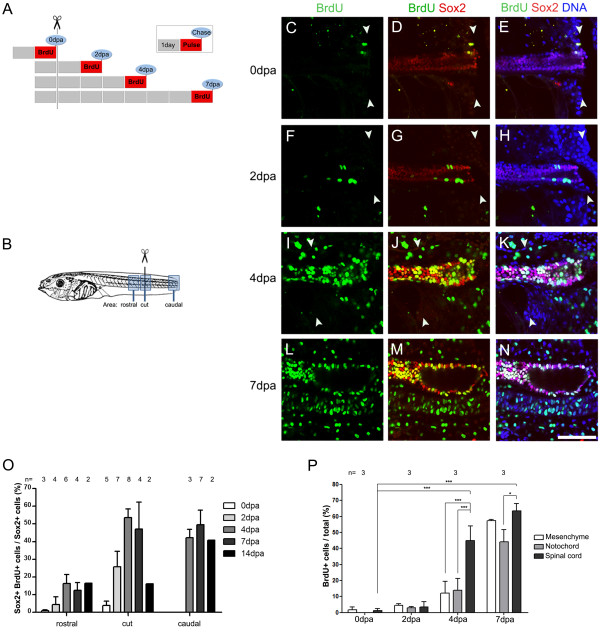
**Tail amputation increases proliferation of Sox2**^**+**^**cells. (A)** Diagram showing the experimental set up to evaluate incorporation of 5-bromo-2'-deoxyuridine (BrdU) after tail amputation of stage 49 tadpoles. Each box represents 1 day, red boxes indicate BrdU incubation (pulse) and blue circles the day of tail fixation (chase). Tadpoles were incubated in BrdU for 24 h chased at different days post amputation (dpa). Then they were processed for coimmunostaining against Sox2 and BrdU and analyzed as described in (C-P). **(B)** Diagram of the rostral, cut and caudal regions analyzed in (C-O). **(C-N)** Sagittal optical section of whole-mount immunofluorescence against BrdU (green) and Sox2 (red) double immunofluorescence from tails labeled as depicted in (A) and fixed at (C-E) 0, (F-H) 2, (I-K) 4 and (L-N) 7 dpa in the cut region (C-K) and caudal region (L-N). DNA is stained in blue. During regeneration, an increase of Sox2^+^/BrdU^+^ cells was observed in the spinal cord and (I-N) neural ampulla. Arrowheads indicate the amputation plane. **(O)** Graph describing the percentage of Sox2^+^/BrdU^+^ cells in the overall Sox2^+^ cells at proximal, cut and caudal regions of spinal cord at different dpa. An increase Sox2^+^ cell proliferation was observed in all the regions mostly at 4 to 7 dpa. **(P)** Graph showing the percentage of BrdU^+^ cells in the spinal cord, notochord and mesenchymal cells at different days post amputation quantified at the cut level. (O,P) The number of tadpoles counted for each point (n) is indicated above the bars. Scale bar: 25 μm.

The spinal cord from amputated larvae contains multiple layers of Sox2^+^ cells (Figure 1I, J) suggesting that tail amputation induces proliferation of Sox2^+^ cells. To address this possibility, BrdU and Sox2 double labeling was performed. Non-amputated and amputated larvae at different dpa were incubated with BrdU for 24 h, fixed at different days post amputation and processed for BrdU and Sox2 double immunostaining (Figure 3A). Colocalization of BrdU/Sox2 in cell nuclei was detected by confocal microscopy on the amputation site and in domains rostral and caudal to the amputation site (Figure 3B).

Basal levels of BrdU incorporation were detected before amputation (Figure 3C-E and O). At 2 dpa, proliferation of Sox2^+^cells in the spinal cord rostral and close to the amputation plane was detected (Figure 3F-H and O). On the fourth day of regeneration, approximately 20% to 60% of the Sox2^+^ cells were proliferating (Figure 3I-K and O). This was not only observed in the amputation region itself, but also rostral to the amputation plane and in the growing tail (caudal region, Figure 3O). This indicates that amputation results in a global activation of proliferation, in agreement with the systemic Sox2 upregulation results. BrdU incorporation persists until 14 dpa, when it first starts to decrease in the area surrounding the amputation plane (Figure 3O). Interestingly, during early phases of regeneration most BrdU^+^ cells of the spinal cord were Sox2^+^, and Sox2^-^/BrdU^+^ cells only appeared at 4 and 7 dpa ( Additional file 2A). Although these results showed that most Sox2^+^ cells start a proliferative cycle it is not possible to discard that some of the proliferating cells were Sox2 negative before damage and then start to express Sox2 and enter into a proliferative stage. Double staining of BrdU^+^ cells with proliferating cell nuclear antigen (PCNA) confirmed that BrdU incorporation corresponds to cells entering the cell cycle and not because of DNA damage (see arrows in Additional file 2B).

Interestingly, at 4 dpa significant increase of BrdU incorporation occurs in spinal cord resident cells, and only low levels of proliferation are detected in the notochord and the mesenchyme surrounding the spinal cord (Figure 3P). Only at 7 dpa BrdU incorporation in the notochord and mesenchyme approach the levels observed in the spinal cord (Figure 3P). In conclusion, tail amputation activates cell proliferation, being initially upregulated in cells along the spinal cord, followed by notochord and mesenchymal cells.

### Overexpression of a dominant negative form of Sox2 impairs tail regeneration in *Xenopus laevis*

Transgenesis was performed to investigate the role of Sox2 in spinal cord regeneration. We used a predicted dominant negative construct lacking most of the Sox2 DNA-binding domain, fused to the glucocorticoid receptor (Sox2BD(−)-human glucocorticoid receptor (GR)) to control nuclear translocation and Sox2 activity, by addition of exogenous dexamethasone. An effect of this construct on other members of the SoxB1 family cannot be discarded. This construct has been used to block Sox2 function during early *Xenopus* development [20], and a similar construct (Sox2BD(−)) reproduces the phenotypes observed with a different dominant negative construct of Sox2 (Sox2EnR) [20,28]. To regulate Sox2BD(−)-GR transcription, its expression was controlled under the heat-shock (HS) inducible promoter (see Additional file 3A). F0 transgenic tadpoles were prepared using the meganuclease method with either HS::enhanced green fluorescent protein (EGFP) (negative control) or a mixture of HS::Sox2BD(−)-GR and HS::EGFP Tadpoles were raised until stage 42 or 49, heat shocked daily, and EGFP-positive tadpoles selected for further analysis. *In situ* hybridization against the glucocorticoid receptor showed that 95% (55/58) of EGFP-positive tadpoles were positive for the Sox2BD(−)-GR transgene ( Additional file 3B).

**Figure 4:**
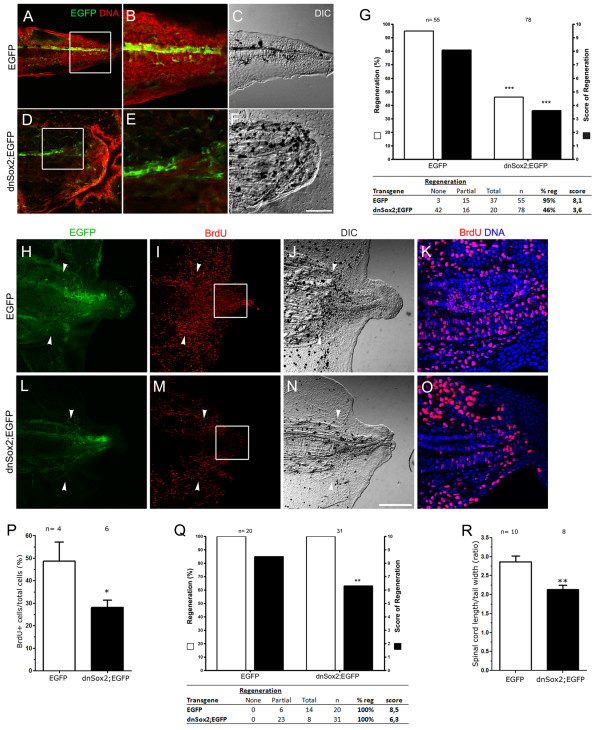
**Overexpression of a dominant negative form of Sox2 impairs tail regeneration. (A-F)** Sagittal optical sections showing the predominant phenotype of regenerated tails in (A-C) enhanced green fluorescent protein (EGFP; total regeneration) and (D-F), dnSox2; EGFP (no regeneration) F0 transgenic tadpoles amputated at stage 42. (A,B,D,E) Images showing transgenic EGFP expression (green) and DNA (red) staining. (B,E) Higher magnifications from the areas indicated in (A) and (D), respectively. (C,F) Differential interference contrast (DIC) microscopy from (A) and (D), respectively. **(G)** Graph showing the regenerative efficiency of EGFP and dnSox2; EGFP stage 42 transgenic tadpoles. White bars represent the percentage of regeneration and black bars the score of regeneration. dnSox2; EGFP tadpoles have a decrease of regenerative efficiency compared to EGFP. **(H-O)** 5-Bromo-2'-deoxyuridine (BrdU) incorporation in (H-K) EGFP and (L-O) dnSox2;EGFP F0 transgenic stage 49 tadpoles at 4 days post amputation (dpa) and (J,N) DIC microscopy from tails depicted in (H) and (L). (K-O) Magnification of the zone labeled in (I) and (M). **(P)** Graph showing the percentage of BrdU^+^ cells in the spinal cord from high EGFP expressing transgenic tadpoles represented in (K) and (O). BrdU incorporation was diminished in dnSox2; EGFP compared to EGFP controls. **(Q)** Regenerative efficiency of EGFP and dnSox2; EGFP transgenic tadpoles amputated at stage 48, a decrease in the score of regeneration was observed in dnSox2; EGFP. **(R)** Graph showing that the regenerated spinal cord length of dnSox2; EGFP transgenic tadpoles decreased compared to controls (EGFP). The length of the spinal cord was corrected by the tail width. (G,P-R) The number of tadpoles analyzed (n) is indicated above the bars. Scale bars: 50 μm.

To evaluate the effect of overexpressing this construct on tail regeneration, F0 transgenic tadpoles, either HS::EGFP (hereafter referred as EGFP) or HS::Sox2BD(−)-GR HS::EGFP (hereafter referred as dnSox2;EGFP) at stage 42 were tail amputated, heat shocked and incubated with dexamethasone daily until 6 dpa. At this stage 95% of the EGFP tadpoles regenerated their tails with an average score of 8.1. In contrast, dnSox2 tadpoles showed disrupted tail regeneration and only 46% of them regenerated the tail with a score of 3.6 (Figure 4A-G). Furthermore, when dnSox2;EGFP tadpoles were separated into groups of low, medium and high levels of EGFP expression, the effect on tail regeneration was dose-dependent, establishing a clear inverse correlation between dnSox2;EGFP activity and tail regeneration (see Additional file 3C). Interestingly, among 55 tadpoles analyzed, 89% (8/9) of those expressing dnSox2;EGFP in the dorsal region of the spinal cord regenerated their tail (see Additional file 3D), whereas only 18.2% (2/11) of the tadpoles that express dnSox2; EGFP in the ventral region regenerated (data not shown). This is in line with the observation that Sox2 levels are lower in the dorsal side of the ependymal tube (Figure 1A). In conclusion, at stage 42 overexpression of the dnSox2 construct impairs tail regeneration.

Inhibition of Sox2 or Sox3 levels during spinal cord development in chick embryos promotes cell cycle exit of neural progenitors [21,22]. Similarly, Sox2 deficiency in adult mice impairs proliferation of NSC in the dentate gyrus [18]. Hence, we decided to test the effect of the dnSox2 on cell proliferation after tail amputation. The tail was amputated in EGFP and dnSox2;EGFP F0 transgenic tadpoles at stage 49, heat shocked daily, incubated with dexamethasone and exposed to BrdU for 24 h until 4dpa. BrdU incorporation levels were reduced in the spinal cord tissue of dnSox2;EGFP tadpoles (Figure 4H-P). Remarkably, BrdU incorporation was also affected in the notochord, but not in mesenchymal cells (Figure 4K,O). Overexpression of dnSox2 at stage 49 only affected the quality of the regenerated tails (a statistically significant reduction in the regeneration score), but did not have an effect on the percentage of regeneration (Figure 4Q). Nevertheless, detailed analysis showed that the length of the spinal cord was reduced in dnSox2;EGFP transgenics when compared to EGFP transgenic controls (Figure 4R).

### Spinal cord regeneration and Sox2 expression during metamorphosis

It has previously been reported that spinal cord transection of *Xenopus laevis* larvae at the level of the fifth body segment has different effects depending upon metamorphic stages. Before metamorphic climax, larvae have the ability to recover coordinated swimming and behavior, while at the end of metamorphosis (stage 66), froglets lose this ability [7,10,29]. In order to have a better model system to study spinal cord regeneration, we set up spinal cord transection in *Xenopus* larvae at different metamorphic stages.

**Figure 5:**
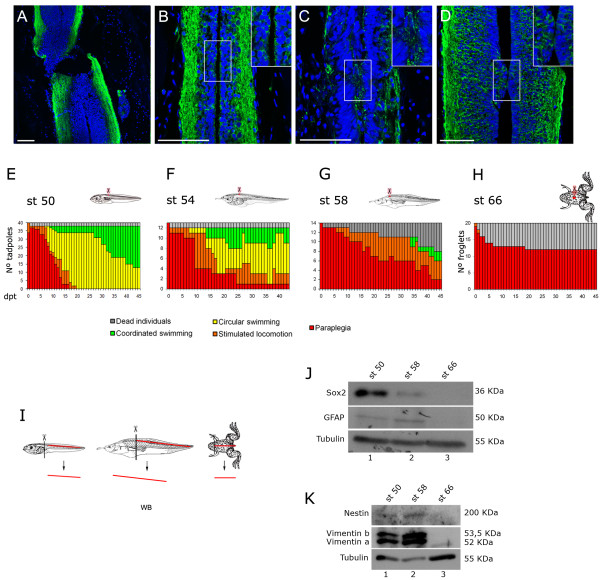
**Spinal cord regeneration and Sox2 expression during metamorphosis. (A-D)** Immunofluorescences of stage 50 tadpoles against acetylated α-tubulin (green) on horizontal sections from the injured area at (A) 0 days post transection (dpt) with paraplegia phenotype, (B) 0 days post sham operation (dps) that has coordinated swimming, (C) 7 dpt with circular swimming and (D) 28 dpt with coordinated swimming. Insets in (B-D) show 1.5 times magnifications of the areas contained in the frames, which correspond to the transection levels. **(E-H)** Graphs showing functional recovery of tadpoles transected at different metamorphic stages (st). Functional recovery decreases meanwhile metamorphosis undergoes. **(I)** Diagram depicting the isolation of spinal cord samples used for J,K. **(J,K)** Western blot analysis of neural-related markers (J) Sox2, glial fibrillary acidic protein (GFAP), (K) Nestin and Vimentin at different stages of metamorphosis. Scale bars: 50 μm.

To this end, we performed complete spinal cord transection at the mid-thoracic level in anesthetized prometamorphic larvae (stage 50). As controls, larvae were sham operated with an incision in the dorsal skin and skeletal muscle, leaving the spinal cord unharmed. Transection resulted in a complete discontinuity of the ependymal canal, generating rostral and caudal stumps separated by an ablation gap, with total disruption of axonal tracts (Figure 5A,B and Additional file 4A,B). Functional recovery of operated tadpoles was evaluated on a daily basis for 8 weeks. After recovery from anesthesia, all larvae had a ‘paraplegia’ phenotype (Figure 5E, see Methods for a detailed description of each behavioral category). Tadpoles were not able to move except for a reflex movement of the head and oral tentacles when touched rostral to the transection site ( Additional file 5 and Additional file 6). At 15 days post transection (dpt), most animals showed stimulated locomotion or circular swimming, indicating a partial recovery of locomotion (Figure 5E, and Additional file 7 and Additional file 8). Finally, at 7 weeks post transection most have regained coordinated swimming with a continuous anteroposterior movement wave (Figure 5E, and Additional file 9). No paraplegia phenotypes were observed in sham-operated animals, and few animals died due to the procedure ( Additional file 4C).

Functional recovery was analyzed concurrently with the anatomical and histological condition of the spinal cord during the regenerative period. Anti-acetylated α-tubulin immunofluorescence and nuclei staining at 7 dpt demonstrated a partial continuity of the ependymal canal, and some axonal tracts are observed in the transected region at least on one side of the spinal cord (Figure 5C). At 28 dpt the ependymal epithelium and canal showed almost normal continuity and axonal tracts were re-established (Figure 5D). One explanation for this regeneration is an axonal regeneration-based mechanism, but we cannot discard that reestablishment of the ependymal epithelium may contribute to spinal cord regeneration after transection.

Functional recovery after spinal cord transection was also analyzed in premetamorphic animals (stage 54), and at the beginning and end of metamorphic climax (stage 58 and 66, respectively). We found that while stage 54 tadpoles are able to recover (Figure 5F), this capacity is abruptly reduced after the onset of metamorphic climax at stage 58 (Figure 5G). Furthermore, juvenile froglets (stage 66), which have concluded metamorphosis, completely lack the ability to regenerate from paraplegia (Figure 5H and Additional file 10, Additional file 11, and Additional file 12). Approximately 40% of the transected animals at metamorphic climax stages died. This effect was not observed in sham-operated animals ( Additional file 4D,E), suggesting that death is a consequence of the inability of these animals to recover from spinal cord injury. From these results, we envision that spinal cord transection of *Xenopus* tadpoles at different metamorphic stages should provide a robust experimental paradigm to understand the molecular, genetic and cellular mechanisms underlying spinal cord regeneration.

In order to understand the lost of regenerative capabilities during metamorphosis, Sox2 and other NPC and ependymal cell markers, such as glial fibrillary acidic protein (GFAP), Vimentin and Nestin, were analyzed in *Xenopus* larvae spinal cord at different stages throughout metamorphosis. For this, larvae spinal cords were isolated at different stages as described [30], homogenized and analyzed by western blotting (Figure 5I-K). Interestingly, western blot analysis showed high Sox2 protein levels at stage 50, whereas a weak signal was observed at stage 58 (compare lanes 1 and 2, Figure 5J), this is concomitant with the abrupt reduction in functional recovery from spinal cord transection at stage 58 (Figure 5G). Conversely, similar protein levels for GFAP, Vimentin and Nestin were observed between stage 50 and 58 (compare lanes 1 and 2 from Figure 5J,K). However, all these proteins were not detected in non-regenerative froglet spinal cord (Figure 5J,K, stage 66 lane 3). Thus, Sox2 protein levels showed a clear correlation with functional recovery after spinal cord transection, suggesting its possible relevance in this process.

### Response of Sox2^+^ cells to spinal cord transection

It has been proposed that ependymal cells from the rostral and caudal stumps are activated and migrate to the ablation gap in response to spinal cord transection [7,15]. Based upon this data, we decided to test the effect of transection on Sox2^+^ cells. For this purpose, sham-operated or transected stage 50 tadpoles were incubated with BrdU for 2 days, starting at 2 days after surgery, followed by fixation and BrdU/Sox2 double labeling in horizontal sections.

**Figure 6:**
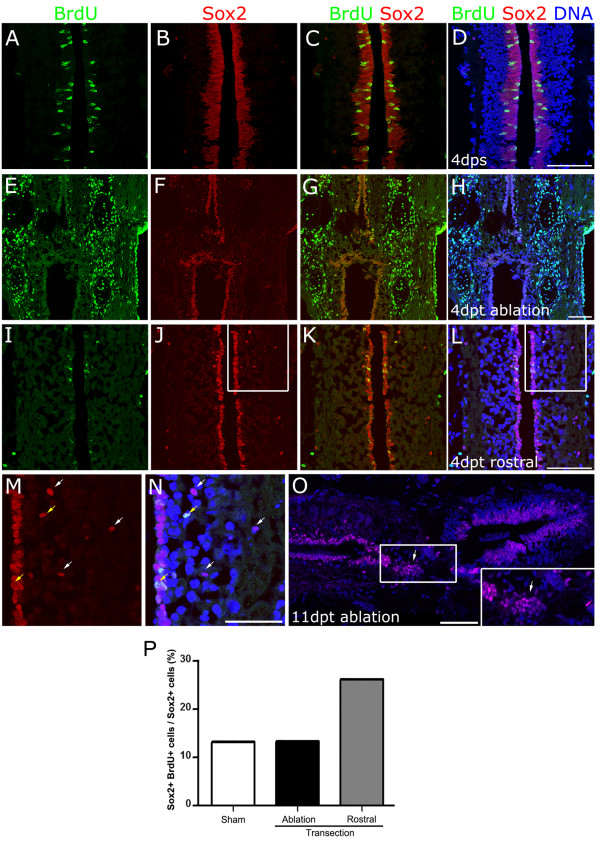
**Response of Sox2**^**+**^**cells to spinal cord transection. (A-L)** Tadpoles were incubated with 5-bromo-2'-deoxyuridine (BrdU) between days 2 and 4 after (A-D) sham operation or (E-L) spinal cord transection. Horizontal sections from animals fixed at 4 days post sham operation (dps) or days post transection (dpt) were immunostained for BrdU (green), Sox2 (red) and DNA (blue). (E-H) Transection zone and (I-L) region rostral to transection zone. **(M,N)** Magnification of (J) and (L). White arrows indicate Sox2^+^/BrdU^-^ cells distant to ependyma, and Sox2^+^/BrdU^+^ cells are indicated by yellow arrows. **(O)** Sox2 immunofluorescence on a horizontal section from tadpoles at 11 dpt. Inset: magnification of the ablation gap that contains Sox2^+^ cells aggregates (arrows). **(P)** Analysis of the percentage of Sox2^+^/BrdU^+^ over total number of Sox2^+^ cells from samples represented in A-L. Sox2^+^ cell proliferation increase at the rostral level of the transection site. Scale bars: (D,H,L) 50 μm, (N,O) 25 μm.

In non-transected animals, Sox2^+^/BrdU^+^ cells were almost exclusively found in cells with elongated nuclei located in the ependymal layer lining the central canal (Figure 6A-D). As expected, we observed a robust increase on BrdU^+^ cells in the tissues surrounding the transection site (Figure 6E-H). Contrary to that, we found a reduction in the number of Sox2^+^/BrdU^+^ cells located in the ependymal canal at the transection zone (Figure 6E-H). Interestingly, the total amount of Sox2^+^ cells lining the ependymal canal was also strongly reduced, and its nuclear shape changed from elongated to round (Figure 6, compare (F) with (B)). Although the number of total Sox2^+^ cells and Sox2^+^/BrdU^+^ cell decrease after transection, we have found that the ratio of proliferating Sox2^+^/BrdU^+^ cells over the total number of Sox2^+^ cells did not change in the ablation gap area and tend to increase in the rostral region (Figure 6I-L) compared to sham controls (Figure 6P).

Furthermore, the main histology of the tissue lining the central canal was altered after transection. In non-transected animals, spinal cord cells are very well organized and packed, with the ependymal canal lined by Sox2^+^ elongated nucleus, followed by a layer of round nuclei that is Sox2 negative (Figure 6D). On the contrary, after transection ependymal layer of elongated nucleus was reduced to a one-cell layer of round Sox2^+^ nucleus, clearly observed at the rostral level (Figure 6L,M). In transected animal spinal cords, but not in those from sham-operated animals, many round Sox2^+^ nucleus, most of them BrdU negative, were found distant to the central canal (see white arrows in Figure 6M,N) meanwhile Sox2^+^/BrdU^+^ cells are found in the ependymal epithelium or closer to it (see yellow arrows in Figure 6M,N).

These changes in the localization and morphology of Sox2^+^ cells after transection, in agreement with their proliferative behavior, suggest a possible role of Sox2^+^ cell migration from distant regions to the ablation gap during spinal cord regeneration.

Sox2 staining was also performed in stage 50 tadpoles at 11 dpt. A partially recovered central canal surrounded by Sox2^+^ cells and some supernumerary canals were detected in transverse sections (see arrow and arrowhead in Additional file 13A,B). Horizontal sections showed that Sox2^+^ cell aggregates were colonizing the space between the spinal cord stumps (see arrow in Figure 6O).

These results suggest that transection induces Sox2^+^ cells lining the ependymal canal to migrate into the ablation gap. Although, we were expecting to see an increase in the proliferation of Sox2^+^ cells, we found the opposite result: a reduction in the number of Sox2^+^/BrdU^+^ cells. We propose two possible explanations for these results: (i) Sox2^+^ cells were activated very rapidly and DNA replication occurred during the first 2 days after transection, before BrdU addition, or (ii) it is plausible that no massive proliferation of Sox2^+^ cells is necessary and that in response to injury, the cells lining the ependymal canal change their shape and migrate, explaining the decrease of Sox2^+^ cells in the ependyma.

## Discussion

In the present study we explored the behavior of Sox2^+^ cells in spinal cord regeneration after tail amputation and spinal cord transection of *Xenopus* larvae. Either type of injury results in dramatic changes in Sox2^+^ cells. Tail amputation results in increased levels of Sox2 mRNA and protein levels, and also in an augmentation of the overall number of Sox2^+^ cells. This increase occurred in spinal cord regions located up to 5 to 8 mm from the injury site, but was also observed in the lateral line and the olfactory epithelium region, indicating that amputation induces a systemic activation of Sox2^+^ cells. Overexpression of dnSox2 diminished proliferation of spinal cord resident cells disrupting spinal cord and tail regeneration. Moreover, Sox2 levels correlate with regenerative capabilities during metamorphosis and Sox2^+^ cell aggregates repopulate the ablation gap after spinal cord transection. Altogether, these results suggest that Sox2^+^ cells contribute to spinal cord regeneration. This leads to a model in which spinal cord damage activates Sox2^+^ cells proliferation and/or migration that will build-up the spinal cord in the regenerating tail, or reconstitute the ependymal canal after spinal cord transection.

In the transection model system, at 4 dpt a reduction of Sox2^+^ cells lining the ependymal canal was observed, concomitant with the emergence of Sox2^+^ cells in the ablation gap at 11 dpt.

After tail amputation the main change of Sox2^+^ cell behavior is the increase of their proliferation and the total number of cell that might allow the growth of a new ependymal canal. In contrast, in the transection model, the main mechanism of regeneration appears to be the migration of Sox2^+^ cells from distant regions to the injury site restoring spinal cord continuity. In addition, in both models rostral Sox2^+^ cells appears to increase their proliferation in response to injury, suggesting that distant tissues to the lesion also participate in the regenerative process. These suggest that although both processes are different some of the underlying cellular and molecular mechanisms are similar.

### Role of the spinal cord in tail regeneration

Intriguingly, overexpression of dnSox2 influenced the entire tail regeneration program. These results suggest that activation of Sox2^+^ cells is required for regeneration of all tail tissues, instead of solely affecting the spinal cord where Sox2 is expressed. Accordingly, we have found that induction of *sox2* mRNA precedes the increase of notochord progenitor marker (*Xbra*), and BrdU incorporation in spinal cord cells is upregulated before than notochord and mesenchymal cells do. Furthermore, overexpression of dnSox2 primarily affects proliferation of spinal cord resident cells, but also influenced BrdU incorporation in notochord cells (Figure 4O).

This apparent paradox could be explained if the regeneration of the spinal cord is the initial step in tail regeneration, and is hence required for proper regeneration of other tail tissues. This is supported by classical studies in axolotls where removal of the spinal cord suppresses tail regeneration, while transplantation of the spinal cord to a fin results in the formation of a tail with all its axial structures [31]. In addition, detailed observation of the regenerating tail in salamanders showed that spinal cord regenerates before the other tissues [32]. Similarly, contact between the regenerating spinal cord and the wound epithelium induces epimorphic regeneration of the tail [8]. Recently, the spinal cord requirement was demonstrated by studying the effect of surgical spinal cord ablation in *Xenopus* larvae tail regeneration [30]. Our findings now suggest that spinal cord regeneration, on which Sox2^+^ cells are activated, is crucial for tail regeneration in *Xenopus* larvae.

### *Xenopus* tail regeneration proceeds via activation of tissue-specific progenitor cells

Lineage tracing analyses have demonstrated that each tissue in the regenerated tail originates from the same tissue in the regenerative bud, suggesting that activation of tissue-specific stem/progenitor cells is required [11,13,14]. A role for Pax7^+^ muscle satellite cells during skeletal muscle regeneration has been demonstrated [12]. The high percentage of Sox2^+^/BrdU^+^ cells in the regenerated tail and the phenotype obtained by overexpression of a dominant negative Sox2 construct might imply that Sox2^+^ cells are the main source of cellular progenitors required for spinal cord regeneration. Importantly, this mechanism may resemble that involved in homeostatic regeneration in mammals, supporting the notion that *Xenopus* tail regeneration is an excellent model system to study the biology of stem/progenitor cells.

One intriguing observation is that systemic upregulation of *sox2* expression levels and activation of Sox2^+^ cells occur in response to spinal cord damage. It is possible that such a global activation of progenitor cells prepares the whole organism to varying regenerative demands. Several examples of tissues and stem/progenitor cells responding at a distance to systemic signaling activation have been described [33,34]. Identifying the signals involved in such global activation is of great interest for regenerative biology. Prominent evolutionarily conserved candidates are members of the insulin family. In *Drosophila,* insulin regulates ovarian stem cell physiology and insulin-like growth factor 1 (IGF-1) is required for global activation of neural progenitors in response to focal cerebral ischemia in rodents [35]. However, whether insulin-like factors are responsible for the effects observed here are open questions that need to be addressed.

### Role of Sox2^+^ cells in spinal cord regeneration

Accordingly with the results on this work, brain and spinal cord injury in *Xenopus* larvae [36], adult teleost fish [37-39], eel [40] and urodeles [8,41,42] induce proliferation of ependymal cells in the ventricular layer and ependymal cells lining the central canal. While these experiments demonstrate that nervous system damage activates proliferation of neural progenitors in regenerative model systems, they do not provide definitive proof of the need for neural progenitors to achieve successful neural tissue regeneration. Here, we demonstrated that overexpression of dnSox2 reduces the proliferation of spinal cord cells and impairs the proper spinal cord and tail regeneration in *Xenopus* larvae, suggesting that activation of Sox2^+^ cells is necessary for spinal cord and tail regeneration. We observed a reduced effect on tail regeneration at later stages that could be explained because of the high levels of transgene mosaicism when larvae are raised until stage 49. It is important to mention that the dominant negative construct used could also interfere with the activity of other members of the SoxB1 subfamily including Sox1 and 3. Accordingly, without experiments using a more specific tool it is not possible to discard that the effects obtained could be explained by interfering with members of the SoxB1 subfamily.

We envision that activated Sox2^+^ cells could mediate spinal cord regeneration through at least two mechanisms: reconstitution of the ependymal canal via self-renewal of Sox2^+^ cells and/or through neurogenesis by the formation of new neurons and glial cells. Our findings suggest that Sox2^+^ cells are activated after SCI and divide to regenerate the ependymal canal. The fact that new neurons are produced in response to damage in zebrafish spinal cord [37,43] and newt midbrain [42], together with the existence of a secondary wave of neurogenesis during metamorphosis to allow nervous system remodeling [44], support the possibility that the production of new neurons could play a role in spinal cord regeneration in *Xenopus*.

In addition to the mechanism described above, we propose that Sox2^+^ cells can contribute to spinal cord regeneration by a different mechanism. Histological analysis after spinal cord transection in *Xenopus* and salamanders showed outgrowth and migration of ependymal cells from the rostral and caudal stumps toward the ablated gap, allowing the formation of an ependymal bridge [7,8,15]. At the ultrastructural level, ependymal processes and regenerating axons make contact, suggesting that ependymal cells provide a pathway for axonal growth and the consequent regeneration of the spinal cord [15,45,46]. Similarly, Sox2^+^ cellular aggregates found in the ablation gap at 11 dpt (Figure 6O) could correspond to ependymal outgrowths that provide the pathway for axonal regeneration, and their ultrastructural characterization requires future analysis. This scenario is highly reminiscent of the formation of a ‘nerve bridge’ required to promote axonal regrowth after peripheral nerve injury [47]. This is caused by the dedifferentiation of Schwann cells to a progenitor/stem cell state and a collective migration into the gap between the nerve stumps. Sox2 is expressed in immature migrating Schwann cells and is required for proper formation of the ‘nerve bridge’ [48,49]. Based upon these data, we hypothesize that Sox2^+^ cells migrate to the ablation gap allowing reconstitution and continuity of the ependymal canal, as well as provide a substrate for axonal regeneration in *Xenopus* larvae.

### Loss of spinal cord regeneration during metamorphosis

Here, we have reintroduced spinal cord transection of *Xenopus* tadpoles as a model system to study spinal cord regeneration [7,9,10]. *Xenopus* tadpoles lose the regenerative capacity after metamorphosis, providing an excellent model to identify the cellular and molecular mechanisms responsible for regeneration. Western blot analysis showed that Sox2 protein levels seem to decrease during metamorphosis and a strong reduction occurs at the beginning of metamorphic climax (stage 58). This decrease is concomitant with a reduction in other neural progenitors markers (Nestin, GFAP, Vimentin) at the end of metamorphosis (stage 66) when the regenerative capabilities are completely lost, suggesting a possible role for neural progenitors in spinal cord regenerative capacity. Interestingly, at stage 58, peak levels of thyroid hormone, the main regulator of metamorphosis in *Xenopus*, have been observed [44], suggesting that T3/T4 hormones can play a role in modulating Sox2 expression.

At least three hypotheses can explain the loss of regeneration after metamorphosis. One possibility is that insufficient numbers of Sox2^+^ cells are activated in response to SCI, thus making it impossible to reconstitute the ependymal canal. Alternatively, these cells could be activated as efficiently as at earlier stages, but because of cell autonomous and/or non-autonomous changes during metamorphosis, they are mainly fated to glia, and no new neurons are produced. The latter is supported by observations in rodents, where SCI induces rapid proliferation and activation of Nestin-expressing neural progenitors that differentiate into glial cells but not into new neurons [50-53]. Only when neural progenitors isolated from adult spinal cord are transplanted into a neurogenic region on the adult brain is differentiation into neurons observed [54]. A third possibility is that Sox2^+^ cells are activated but they are no longer able to migrate to the ablation gap to provide a substrate for axonal growth.

Therefore we believe that studies comparing regenerative and non-regenerative stages in *Xenopus* tadpoles should provide a fertile ground for identifying the underlying mechanisms that explain the loss of spinal cord regenerative capacity. These studies should provide new insights for the understanding of why mammals are not able to regenerate their spinal cord and how we can manipulate them to promote and improve regeneration upon spinal cord injury.

## Conclusions

This study describes the expression of Sox2 during *Xenopus laevis* spinal cord regeneration after tail amputation and transection. Our results demonstrate that Sox2^+^ cells, which have characteristics of neural progenitors, proliferate after spinal cord injury. Overexpression of a dnSox2 construct impairs tail regeneration decreasing spinal cord cell proliferation, suggesting that the function of Sox2 is necessary for proper regeneration. After spinal cord transection, Sox2^+^ cell population colonizes the ablation gap giving continuity to the injured spinal cord. We support a model in which the Sox2^+^ progenitor population is rapidly amplified after tail amputation to regenerate the ablated spinal cord, whereas during spinal cord transection this population may migrate to restore the ependymal epithelium continuity. This hypothesis is supported by the fact that spinal cord regeneration capability decreases during metamorphosis, while Sox2 expression and others neural progenitor markers decrease as well. Further characterization of this progenitor population behavior will give new insight into the mechanisms that govern spinal cord regeneration in amphibians.

## Methods

### Growth and manipulation of *Xenopus laevis*

*Xenopus laevis* embryos were obtained by *in vitro* fertilization and cultured at 21°C in 0.1 × Barth (8.9 mM NaCl; 102 μM KCl; 238.1 μM NaHCO_3_; 1 mM 4-(2-hydroxyethyl)-1-piperazine-ethanesulfonic acid (HEPES); 81.14 μM MgSO_4_; 33.88 μM Ca(NO_3_)_2_; 40.81 μM CaCl_2_, pH 7.6) supplemented with antibiotics (100 μg/ml penicillin and 100 μg/ml streptomycin) until stage 46 [55]. Afterwards, they were raised in tanks (up to four tadpoles/l) with chlorine-free aerated water at 23°C and fed daily with Sera Micron until they reached the required stage.

Tail amputation experiments were performed in *Xenopus laevis* tadpoles at stages 42 or 49 as described [56,57]. Tadpoles were anaesthetized in 0.1% MS 222, placed on a tissue-covered Petri dish, and amputated vertically at the middle of the tail with iridectomy scissors. Regeneration was assessed at 6 to 8 dpa by determining the percentage of regeneration (% regenerated tails/total amount of tails), and the quality of regeneration (mean of individual tail scores). A score of 10 was assigned to total regeneration (straight tails containing all of the main tail tissues), 5 to partial regeneration (crooked tails or lack of some of the main tissues) and 0 to no regeneration (rounded stumps with no evidence of new elongated tissues) [56]. Percentages of regeneration were analyzed using the *χ*^2^ test. For regeneration score the Mann Withney or Kruskal-Wallis test followed by Dunn’s multiple comparison post test were used. Vital dye was achieved by incubation on DASPEI 1 mM.

For spinal cord transection, tadpoles and froglets were anesthetized in 0.1% MS222, placed on an inverted Petri dish covered by gauze. Dorsal tissue was incised in the middle of the thoracic segment until the spinal cord plane was reached. The exposed spinal cord tissue was cross-sectioned using iridectomy scissors. Tissue planes were then relocated at their corresponding place and compression was made until clot formation. For sham operation, only skin and muscle tissues were injured. In froglets, where vertebrae are ossified, spinal cord was accessed throughout the intervertebral discs and complete spinal cord transection was confirmed checking free-end stumps with a hook. Animals were transferred to tanks containing 0.1 × Barth supplemented with antibiotics, and fed daily. Operated froglets were kept in tanks taking care that the water did not cover the frogs’ heads to avoid drowning.

Animal behavior was classified into the following categories: (i) paraplegia: lack of movement caudal to the transection site ( Additional file 6 and Additional file 11, compare to Additional file 5 and Additional file 10), (ii) stimulated locomotion: spatial displacement after touching the animal caudally to the transection site ( Additional file 7), (iii) circular swimming: spontaneous locomotion in circles ( Additional file 8) and (iv) coordinated swimming: spontaneous swimming in coordinated corporal waves ( Additional file 9).

### *In situ* hybridization and immunofluorescence

Whole-mount *in situ* hybridization was performed as described [58] with minor modifications: anti-digoxigenin antibody dilution was used at 1:5,000, and tadpoles at stage 42 and 49 were incubated with proteinase K for 10 and 15 minutes, respectively. Tails after whole-mount *in situ* hybridizations were embedded in Epon and sectioned in ultramicrotome at 3 μm.

The following antibodies or reagents were used: rabbit polyclonal anti-Sox2 (1:200 for sections and 1:500 for whole-mount, (Cell Signalling 2748s), mouse monoclonal anti-acetylated α-tubulin (1:1,000, Sigma T7451), mouse monoclonal anti-BrdU (1:500, Sigma, B2531), AlexaFluor® 488 or 555 (1:500). DNA was stained with TOTO3 in all samples (1:1,000, Molecular Probes T3604).

When working with sections, samples were fixed in 4% PFA for 2 to 3 h at room temperature and (i) cryoprotected in 20% sucrose, embedded in optimal cutting temperature (OCT) compound, frozen and sectioned at 15 μm, or (ii) embedded in paraffin, sectioned at 15 μm and mounted on silanized slides. For morphology analysis, sections were stained with hematoxylin/safranine. To perform cryosection immunofluoresence, samples were rehydrated in phosphate-buffered saline (PBS), blocked for 1 h in 5% fish gelatin, incubated on primary and secondary antibodies for 2 h at room temperature in the same blocking solution, washed for 10 minutes after each antibody incubation, and followed by DNA staining and mounting. For paraffin section immunofluorescence, samples were permeabilized for 10 minutes in PBS containing 0.2% Triton X-100, blocking for 1 h In PBS containing 0.1% Tween 20 and 10% goat serum, incubated overnight at 4°C with primary antibody diluted in the same blocking solution; washed for 10 minutes in PBS containing 0.1% Tween 20, incubated with secondary antibody for 2 h at room temperature, washed similar to above followed by DNA staining and mounting.

For proliferation assays, BrdU was added to the culture medium to a final concentration of 400 μM for the indicated times (see Figures 3,4,5, and 6). Double labeling for Sox2 and BrdU was performed as follows. Tails were fixed in 4% PFA 3 h at RT, permeabilized for 45 minutes in PBS containing 0.5% Triton X-100 (PBS-Tr), incubated in 0.25% trypsin for 15 minutes on ice, and with 4 M HCl for 20 minutes, washed in PBS-Tr and incubated in blocking solution (PBS-Tr containing 10% goat serum and 1% dimethylsulfoxide (DMSO)). Samples were incubated with both antibodies against Sox2 and BrdU in blocking solution for 2 h at room temperature, and washed for 2 h in PBS-Tr. The same incubations were made for secondary antibodies. For whole-mount immunofluorescence against Sox2 and acetylated α-tubulin, a similar protocol was used, although the HCl treatment was omitted. BrdU immunofluorescence on cryosections was performed as described [36] incubating with Sox2 and BrdU primary antibodies at the same time.

For DNA staining, sections were incubated for 10 minutes in TOTO3, and whole mounts were permeabilized in 1%Triton X-100 for 1 h at room temperature, followed by incubation with TOTO3 1:1,000 for 40 to 60 minutes.

Samples were photographed using fluorescence and differential interference contrast (DIC) microscopy on a confocal microscope (FV-1000 Olympus Confocal Laser Scanning Microscope). Intensity was measured using Z-plot analysis and fire LUT pseudocoloring of ImageJ (NIH). Total cell counting, including quantification of colocalization, was performed using the ImageJ (NIH) cell counter plugin. On tail regeneration cell counting was made in an area of 133 μm^2^ in one focal plane for each sample. On transection experiments an area of 200 μm^2^ from three to five spinal cord sections was quantified. Results for intensity were analyzed by two-way analysis of variance (ANOVA) and Bonferroni *post hoc* test, where as cell counting were analyzed by one-way ANOVA and Tukey *post hoc* test. Error bars in all figures are SEM. *P <0.05, **P <0.01, and ***P <0.001.

### RT-PCR and western blot

Total RNA was prepared from 1 mm of the distal tip of the regenerating tail (n = 10–12), or isolated spinal cord (n = 20). Samples were incubated in TRIzol RNA isolation reagent (Invitrogen) and RNA was isolated following the manufacturer procedure. cDNA was synthesized using M-MLV reverse transcriptase (Promega). Sox2 primers were Fw 5′-CCACACGCCGCCTCGATGT-3′ and Rv 5′-TCAGCCCCCAGCCTCTTGC-3′[59]; Xbra primers were Fw 5′-GCTGGAAGTATGTGAATGGAG-3′ and Rv 5′-TTAAGTGCTGTAATCTCTTCA-3′ [60] and EF1α primers were Fw 5-CCTGAACCACCCAGGCCAGATTGGTG-3′and Fw 5-GAGGGTAGTCAGAGAAGCTCTCCACG-3′. RT-PCRs in regenerating tails were performed three times using independent replicates, representative results were included in figures. Western blotting was performed as described [30]. In all, 15 μg of protein extracted from spinal cords at stages 50, 58 and 66 were loaded. Primary antibodies against Nestin and Vimentin (Rat401 and 14 h7, DSHB) were diluted 1:500, whereas anti-GFAP (N-18 sc6171, Santa Cruz) and anti-tubulin were diluted 1:500 and 1:200,000 respectively.

### Transgene constructs and transgenesis

In order to make the construct p*Sce*I-heat shock protein 70 (HSP70)-SOX2 BD(−) GR emaglutinin epitope (HA), a PCR fragment was amplified from pSP64T-SOX2 BD(−)GR-HA (a kind gift from Y Sasai) [20], containing the 5′ end to the internal *Bam*HI site, and then subcloned into pGEMT-easy. Then, to reconstitute the complete Sox2 construct, a restriction fragment *Bam*HI to *Sal*I from pSP64T-SOX2 BD(−) GR HA was ligated into the pGEMT-easy vector containing the 5′ region of Sox2. Finally, the complete SOX2 BD(−)GR-HA cassette was directionally cloned into *Eco*RV/*Sal*I sites of pI*Sce*I-HSP70 [57] downstream of the HSP70 promoter to obtain pI*Sce*I-HSP70::SOX2 BD(−) GR HA.

Transgenesis was performed using the I*Sce*I meganuclease protocol [57,61]. One-cell stage embryos were injected with a mixture of 50 pg each of pI*Sce*I HSP70::SOX2 BD(−) GR HA and pI*Sce*I HSP70::EGFP, or with 100 pg of pI*Sce*I HSP70::EGFP for controls. Embryos were incubated until the four-cell stage at 12°C, and changed afterwards to 16°C. At stage 42 or 49, tails were amputated, and tadpoles (EGFP and EGFP;dnSox2) were heat shocked daily (30 minutes at 34°C) and treated with 10 μM dexamethasone. Both treatments, the heat shock and dexamethasone incubation did not generate significant changes in the regeneration compared to untreated wild types (data not shown). EGFP expression was classified as low when fluorescence was observe in less than half of spinal cord, medium when about half of spinal cord tissue has EGFP expression and high when EGFP-positive tissue was predominant.

## Competing interests

The authors declare that they have no competing interests.

## Authors’ contributions

MG performed most of the experiments. RM, NS, RT, EGC and DLL carried out specific experiments. MG and RM did the preparation of figures. MG and JL conceived the study, made the interpretation of data and wrote the manuscript. All authors read and approved the final manuscript.

## Supplementary Material

Additional file 1 **Sox2 levels are upregulated after tail amputation. (A)** Ultramicrotome sagittal section of 7 dpa tails analyzed by *sox2in situ* hybridization, safranine counterstain. *sox2* is detected in the spinal cord (SC). Notochord is indicated (NC). Arrowheads: amputation plane. **(B,C)** Quantification of Sox2 **(B)** immunofluorescence intensity and **(C)** Sox2^+^ cells number in different regions of the amputated tadpole over the total number of cells in the spinal cord. **(D,E)** Immunofluorescence against **(D)** acetylated α-tubulin (green) in the ampulla region at 4 dpa, cilia are observed (arrow). **(E)** Immunofluorescence against Sox2 in the rostral region. Inset shows Sox2^+^ nuclei with delamination morphology. DNA was stained in blue in D and E. **(F)** RT-PCR analysis of *sox2* mRNA levels from isolated spinal cord of non-amputated tadpoles. Similar levels were found at stage 50 and 56. *EF1α* was used as loading control. Scale bars A: 100 μm; D,E: 50 μm. Click here for file

Additional file 2 **Cell proliferation during tail regeneration. (A)** Percentage of Sox2^+^ and Sox2^-^ cells that incorporated BrdU in the regenerated tail evaluated at cut level. Most of the BrdU incorporating cells were Sox2^+^. **(B)** BrdU (green) and PCNA (red) immunofluorescence on transversal sections of the regenerated tissue in tails at 6dpa. Examples of double positive cells are indicated by arrows. DNA was stained in blue. The number of samples (n) is indicated above the bars. Scale bar: 50 μm. Click here for file

Additional file 3 **Overexpression of a dominant negative form of Sox2 impairs tail regeneration. (A)** Scheme of transgenic tadpoles generation. One-cell embryo was injected with the EGFP alone or plus dnSox2 vector (EGFP; dnSox2), raised until tadpole-stage, tail amputated and exposed to heat-shock (HS) and dexamethasone incubation (DEX). Sequences in the vectors are: restriction sites for the I-SceI meganuclease (I-SceI), *Xenopus* HSP70 promoter, human glucocorticoid receptor (GR), hemaglutinin epitope (HA) and EGFP. **(B)** in situ hybridization of EGFP positive transgenic tadpoles using a GR antisense probe, most of dnSox2;EGFP transgenics were positive for GR detection in contrast to EGFP transgenic tails that were negative for GR. **(C)** Transgenic tadpoles were classified in three classes (low, medium, high) based on estimation of EGFP expression (see Methods). White bars correspond to percentage of regeneration and black bars to score of regeneration. The efficiency of regeneration of dnSox2; EGFP tadpoles decreased according as EGFP expression increase. Number of tadpoles analyzed (n) is indicated above the bars. **(D)** Total regeneration phenotype from transgenic tadpoles expressing dnSox2; EGFP mainly in the dorsal side of the spinal cord. Scale bars: 50 μm. Click here for file

Additional file 4 **Histology of spinal cord transection.** Complete spinal cord transection was performed in *Xenopus laevis* at stage 50, 58 and 60. **(A,B)** Histological appearance was evaluated staining sagittal sections of injured area at stage 50 with hematoxylin/safranin. Lost of continuity is observed at **(A)** 4dpt compared to **(B)** sham control. **(C,D,E)** Phenotype of the sham-operated controls at **(C)** stage 50**, (D)** 58 and **(E)** 66. Scale bar: 100 μm. Click here for file

Additional file 5 **Movie 1.**Tadpole - wild type. Click here for file

Additional file 6 **Movie 2.**Tadpole - paraplegia. Click here for file

Additional file 7 **Movie 3.**Tadpole - stimulated locomotion. Click here for file

Additional file 8 **Movie 4.**Tadpole - circular swimming. Click here for file

Additional file 9 **Movie 5.**Tadpole - coordinated swimming. Click here for file

Additional file 10 **Movie 6.**Froglet - wild type. Click here for file

Additional file 11 **Movie 7.**Froglet - paraplegia at 0dpt. Click here for file

Additional file 12 **Movie 8.**Froglet - paraplegia at 36dpt. Click here for file

Additional file 13 **Sox2 expression during spinal cord regeneration after transection.** Sox2 (red) immunofluorescence on transversal section at 11dpt in the ablation gap area. DNA was stained in blue. Sox2^+^ cells appear to form a main (arrow) and a supernumerary (arrowhead) ependymal canal. Scale bar: 25 μm. Click here for file
