# Supplementary figures and images for: Spinal cord regeneration in Xenopus tadpoles proceeds through activation of Sox2-positive cells
Source: Neural Dev. 2012 Apr 26;7:13. doi: 10.1186/1749-8104-7-13 (PMC3425087; doi:10.1186/1749-8104-7-13)

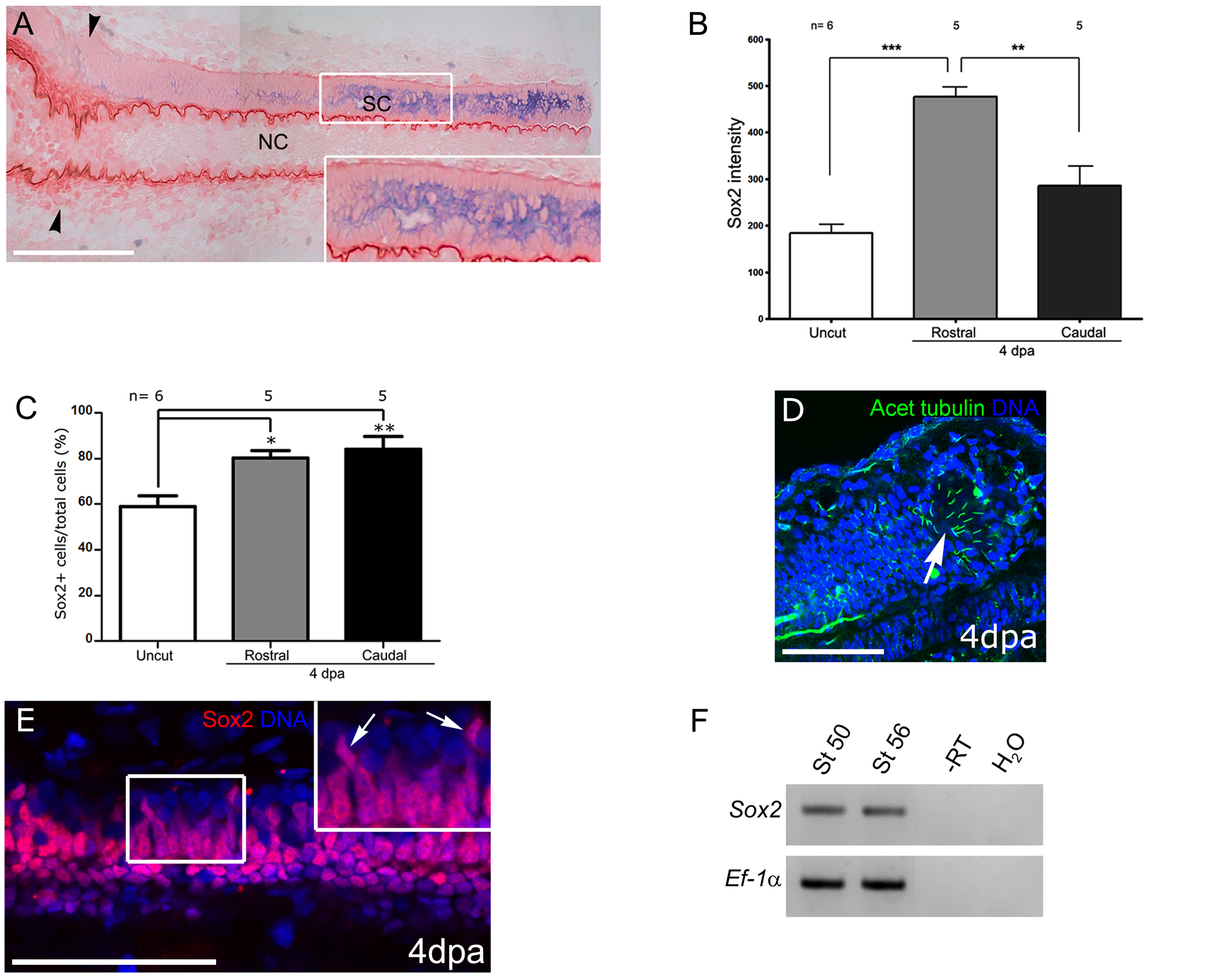

Supplement: Additional file 1 — Sox2 levels are upregulated after tail amputation. (A) Ultramicrotome sagittal section of 7 dpa tails analyzed by sox2in situ hybridization, safranine counterstain. sox2 is detected in the spinal cord (SC). Notochord is indicated (NC). Arrowheads: amputation plane. (B,C) Quantification of Sox2 (B) immunofluorescence intensity and (C) Sox2+ cells number in different regions of the amputated tadpole over the total number of cells in the spinal cord. (D,E) Immunofluorescence against (D) acetylated α-tubulin (green) in the ampulla region at 4 dpa, cilia are observed (arrow). (E) Immunofluorescence against Sox2 in the rostral region. Inset shows Sox2+ nuclei with delamination morphology. DNA was stained in blue in D and E. (F) RT-PCR analysis of sox2 mRNA levels from isolated spinal cord of non-amputated tadpoles. Similar levels were found at stage 50 and 56. EF1α was used as loading control. Scale bars A: 100 μm; D,E: 50 μm. [file 1749-8104-7-13-S1.tiff]

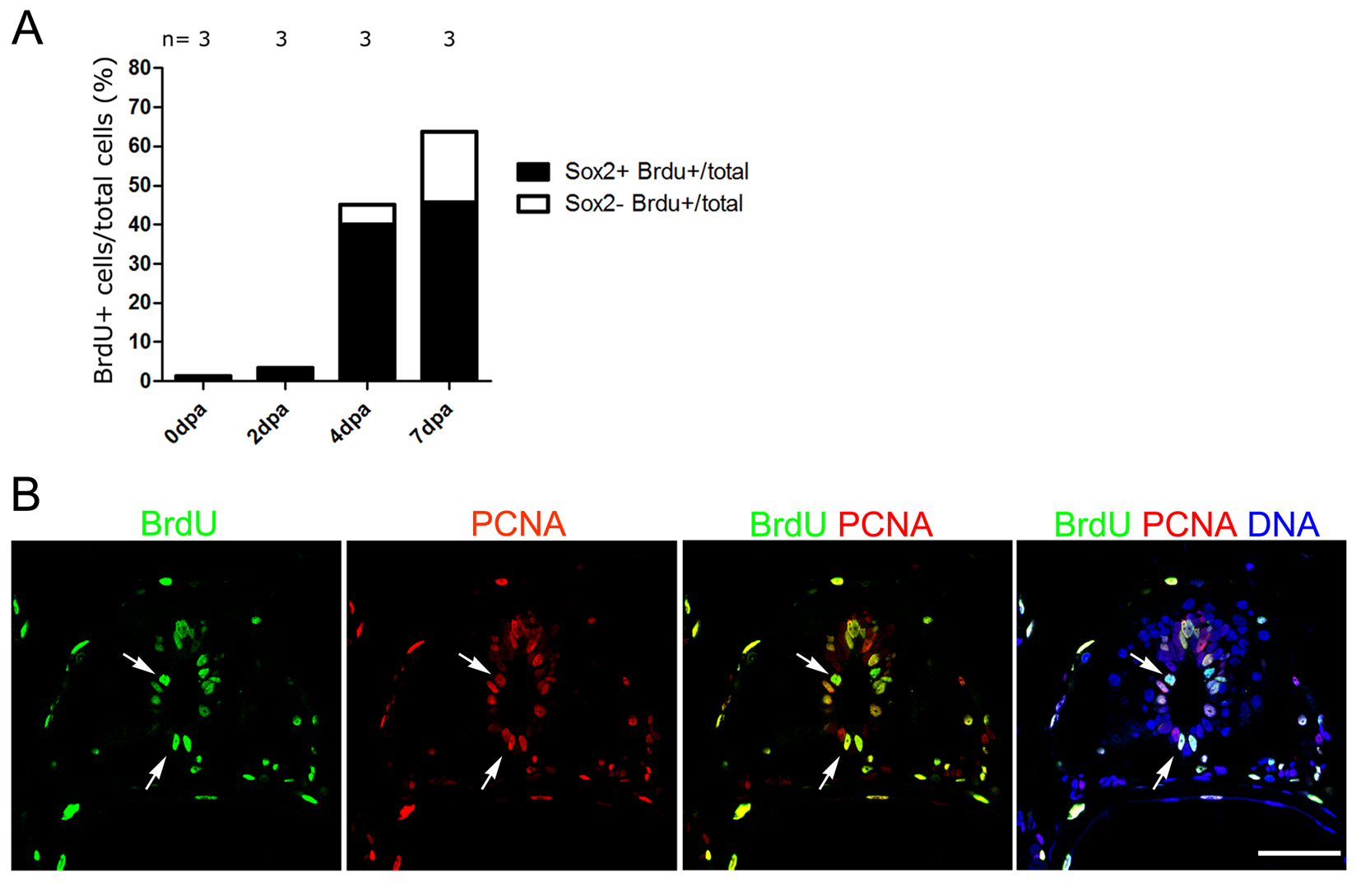

Supplement: Additional file 2 — Cell proliferation during tail regeneration. (A) Percentage of Sox2+ and Sox2- cells that incorporated BrdU in the regenerated tail evaluated at cut level. Most of the BrdU incorporating cells were Sox2+. (B) BrdU (green) and PCNA (red) immunofluorescence on transversal sections of the regenerated tissue in tails at 6dpa. Examples of double positive cells are indicated by arrows. DNA was stained in blue. The number of samples (n) is indicated above the bars. Scale bar: 50 μm. [file 1749-8104-7-13-S2.tiff]

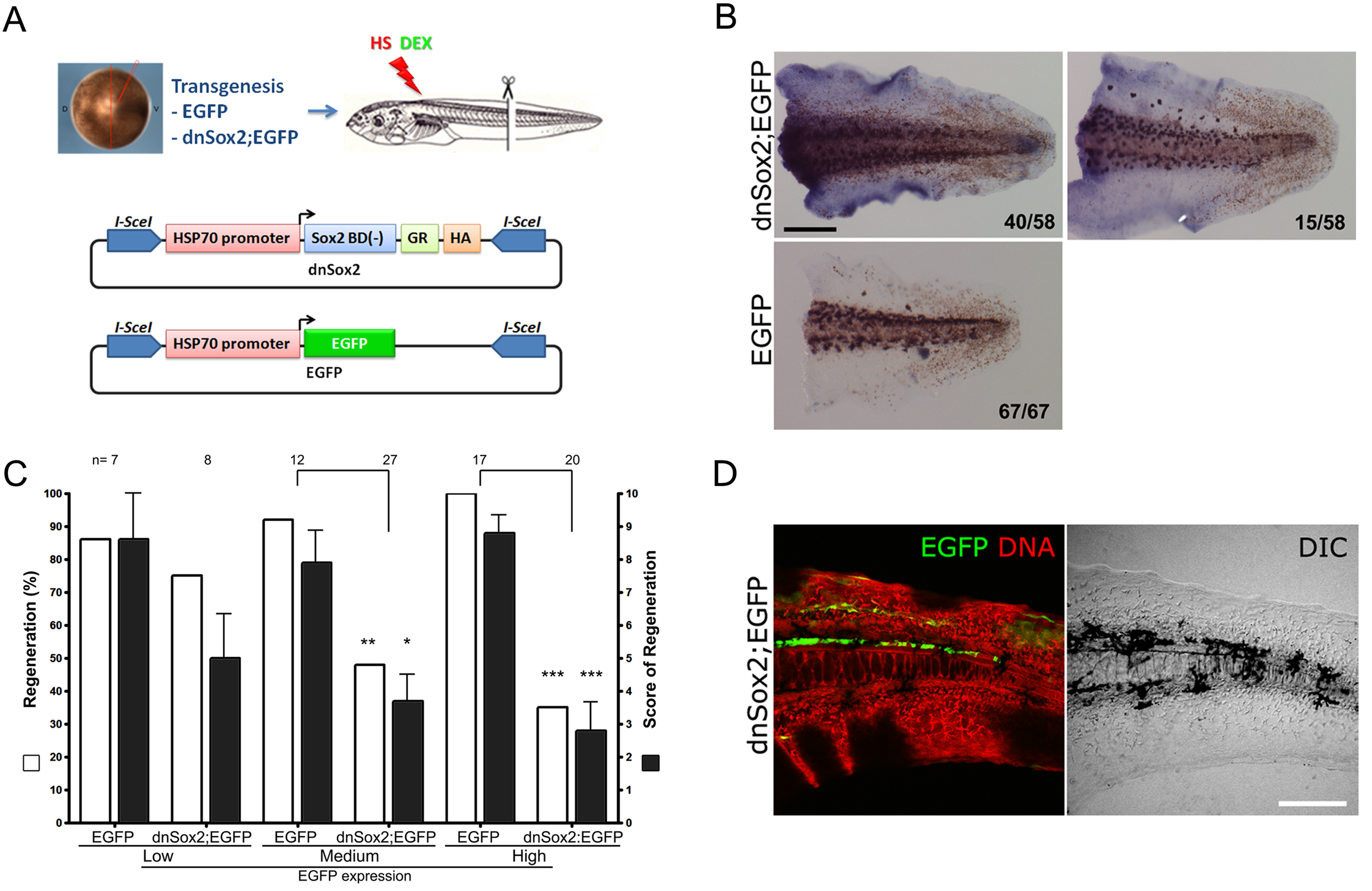

Supplement: Additional file 3 — Overexpression of a dominant negative form of Sox2 impairs tail regeneration. (A) Scheme of transgenic tadpoles generation. One-cell embryo was injected with the EGFP alone or plus dnSox2 vector (EGFP; dnSox2), raised until tadpole-stage, tail amputated and exposed to heat-shock (HS) and dexamethasone incubation (DEX). Sequences in the vectors are: restriction sites for the I-SceI meganuclease (I-SceI), Xenopus HSP70 promoter, human glucocorticoid receptor (GR), hemaglutinin epitope (HA) and EGFP. (B) in situ hybridization of EGFP positive transgenic tadpoles using a GR antisense probe, most of dnSox2;EGFP transgenics were positive for GR detection in contrast to EGFP transgenic tails that were negative for GR. (C) Transgenic tadpoles were classified in three classes (low, medium, high) based on estimation of EGFP expression (see Methods). White bars correspond to percentage of regeneration and black bars to score of regeneration. The efficiency of regeneration of dnSox2; EGFP tadpoles decreased according as EGFP expression increase. Number of tadpoles analyzed (n) is indicated above the bars. (D) Total regeneration phenotype from transgenic tadpoles expressing dnSox2; EGFP mainly in the dorsal side of the spinal cord. Scale bars: 50 μm. [file 1749-8104-7-13-S3.tiff]

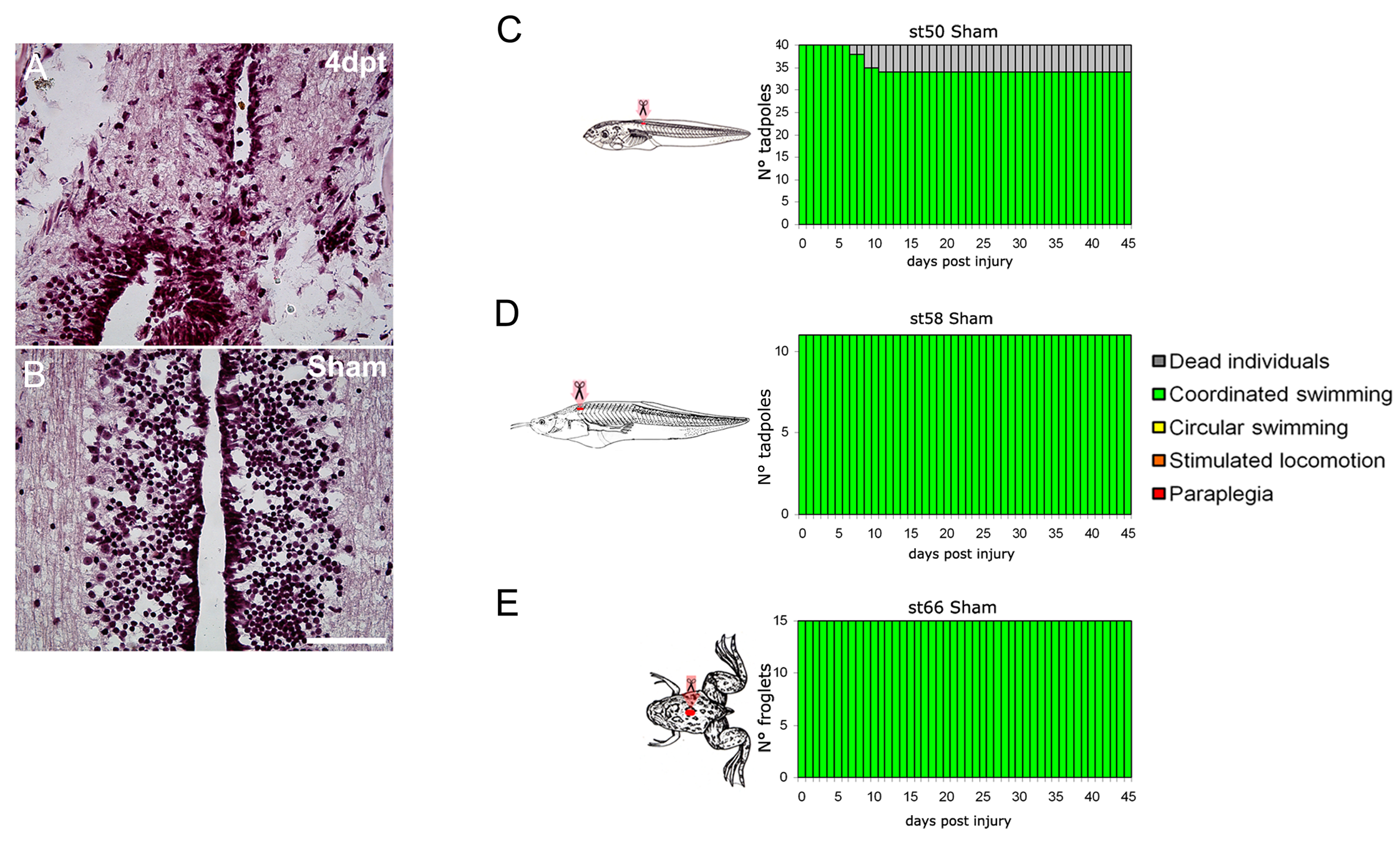

Supplement: Additional file 4 — Histology of spinal cord transection. Complete spinal cord transection was performed in Xenopus laevis at stage 50, 58 and 60. (A,B) Histological appearance was evaluated staining sagittal sections of injured area at stage 50 with hematoxylin/safranin. Lost of continuity is observed at (A) 4dpt compared to (B) sham control. (C,D,E) Phenotype of the sham-operated controls at (C) stage 50, (D) 58 and (E) 66. Scale bar: 100 μm. [file 1749-8104-7-13-S4.tiff]

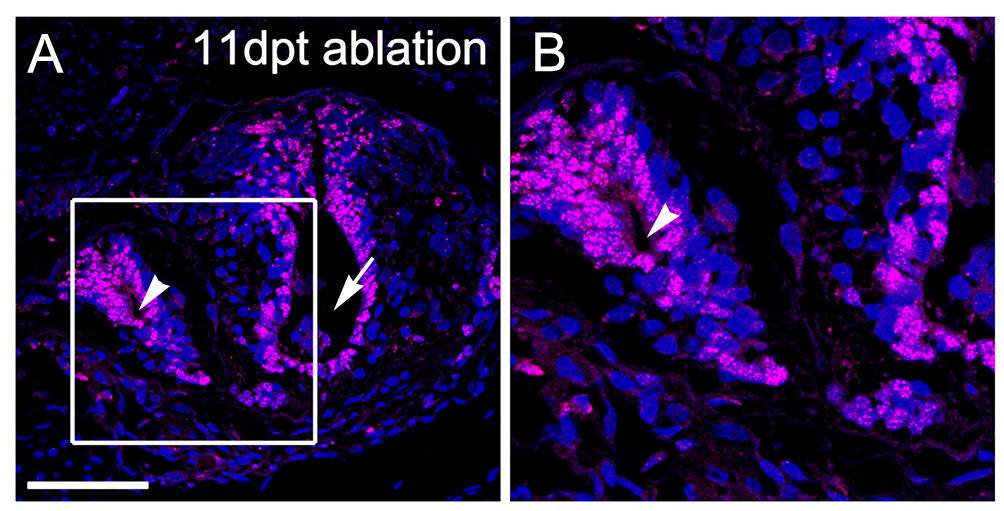

Supplement: Additional file 13 — Sox2 expression during spinal cord regeneration after transection. Sox2 (red) immunofluorescence on transversal section at 11dpt in the ablation gap area. DNA was stained in blue. Sox2+ cells appear to form a main (arrow) and a supernumerary (arrowhead) ependymal canal. Scale bar: 25 μm. [file 1749-8104-7-13-S13.tiff]
